# Chagas disease and social welfare: characterization of the disease in the Brazilian social security and social assistance, 2004-2016

**DOI:** 10.1590/S2237-96222022000200006

**Published:** 2022-06-15

**Authors:** Izabela Lima Perissato, Keile Aparecida Resende Santos, Antônio Marcos Machado de Oliveira, Jean Ezequiel Limongi

**Affiliations:** 1 Universidade Federal de Uberlândia, Curso de Saúde Coletiva, Uberlândia, MG, Brazil; 2 Instituto Nacional do Seguro Social, Agência da Previdência Social de Uberlândia, Uberlândia, MG, Brazil

**Keywords:** Chagas Disease, Social Support, Social Security, Social Welfare, Cross-Sectional Studies

## Abstract

**Objective:**

To characterize the sociodemographic profile of beneficiaries of Brazilian social welfare affected by Chagas disease and identify factors associated with the granting of assistance benefits, 2004 to 2016.

**Methods:**

Cross-sectional study based on secondary data from the Ministry of Labor and Social Security. Logistical regression was performed to estimate crude and adjusted *odds ratios* (OR) and 95% confidence intervals (95%CI).

**Results:**

36,023 benefits were granted; 62.5% were to male; 67.0% to residents of urban areas; 46.7% to residents of Southeast region; 56.7% to people with chronic cardiac form; and 42.7% to the 50-59 age group. Residents of urban areas (OR = 134.9; 95%CI 78.0;233.2), Northeast macro-region (OR = 2.9; 95%CI 2.5;3.1), female (OR = 2.0; 95%CI 1.8;2.1) and age group 60 years or older (OR = 1.6; 95%CI 1,3;1,7) were factors associated with assistance benefits.

**Conclusion:**

Factors related to the area of residence, macro-region, sex and age group increased the chance of granting assistance benefits.

**Study Contributions**

**Main results**

For people with Chagas’ disease, the odds of being granted welfare benefits was higher among residents in urban areas, in the Northeast region, females and those aged 60 or over.

**Implications for services**

The number of people with chronic Chagas’ disease is still high in Brazil. Follow-up of this population in health services is necessary, especially in primary care, aiming to delay disease progression and inability to work.

**Perspectives**

Public health policies for people with chronic Chagas’ disease need to be implemented to promote the quality of life of affected individuals.

## INTRODUCTION

Up until the 1990s, Chagas’ disease was considered the parasitic disease with the greatest socioeconomic impact in Latin America. The burden of the disease, as measured by the “Disability-Adjusted Life Years” (DALY) indicator, was greater than that of all other parasitic diseases combined.^1^ DALY simultaneously measures the effects of mortality and health problems that affect people’s quality of life.^2^ According to the World Health Organization (WHO), approximately 8 million people are currently infected with *Trypanosoma cruzi* worldwide, mainly in Latin America.^3^

Although a large number of infections are asymptomatic, up to 30% of chronically infected people develop cardiac abnormalities and up to 10% develop digestive, neurological or mixed abnormalities, requiring specific treatment. In order to achieve complete cure, treatment must begin in the early stages of infection.^4^

Chagas’ disease causes great biopsychosocial impact on affected individuals, in addition to high costs for health systems, due to the high demand for care and demand for tests, medications, surgical interventions and procedures.^5^ In addition, there are serious economic consequences generated by loss of skilled labor, labor law costs, absenteeism, reduced productivity and social welfare costs.^6^

Article 194 of the 1988 Federal Constitution defines social welfare as “an integrated set of actions at the initiative of the public authorities and society, aimed at ensuring the right to health, the right to social assistance and the right to social security”.^7^ It should be noted that the first two rights are of a non-contributory nature, while social security is eminently contributory in nature.^7^

In Brazil, health is a fundamental right of all people and a duty of the State, guaranteed through social and economic policies.^8^ Social assistance is the social policy that provides for the fulfillment of basic needs, translated into protection for the family, motherhood, childhood, adolescence, old age and the disabled. The Organic Law of Social assistance (Lei Orgânica da Assistência Social - LOAS), No. 8742/93, provides for the organization of social assistance and determines that it must be carried out in an integrated way with sectoral policies, aiming at fighting poverty, guaranteeing minimum social rights, providing conditions to meet social contingencies and universalizing social rights.^9^ Social security, in turn, has mandatory membership, where membership is understood to be the bond established between people who contribute to social security, from which rights and obligations derive. It is a public service, aimed at supporting its members and dependents in situations of social risks or contingencies provided for by law, by granting benefits.^10^

Studies on social welfare concerning specific diseases, containing detailed data, are extremely rare, mainly due to difficulty of access to sources of information.^11^ ^,^ ^12^ With regard to Chagas’ disease in particular, we are not aware of the existence of any national study that addresses the situation of this disease in relation to social welfare. Few studies have analyzed this problem - besides being old publications of studies mostly conducted in the 1970s and 1980s, limited to a small number of cases.^13^ ^-^ ^15^

The number of people with chronic Chagas’ disease is, however, unknown in Brazil. Compulsory notification of these cases has only been established recently, first in the state of Goiás in 2013, followed by Minas Gerais in 2018, and for the entire country in 2020.^16^ ^-^ ^18^ As such, a study of people with Chagas’ disease who are social welfare beneficiaries can contribute to the characterization of the disease in Brazil.

The objective of this study was to characterize the sociodemographic profile of beneficiaries of Brazilian social welfare affected by Chagas’ disease and to identify factors associated with the granting of assistance benefits to them, from 2004 to 2016.

## Methods

This was a cross-sectional study based on data from the Ministry of Labor and Social Security’s Unified Benefits Information System (Sistema Único de Informações de Benefícios - SUIBE). Access to the SUIBE is restricted. It holds sociodemographic data on beneficiaries and data related to the granting of benefits. This system was implemented in mid-2003, but only in 2004 did it start to include a larger number of variables, which is why that year was chosen as the start for data collection. Data access occurred after making a request to the central management level of the National Institute of Social Security (Instituto Nacional do Seguro Social - INSS). The data were made available on an Excel® spreadsheet in January 2017.

We included individuals who received social assistance and social security benefits granted by the INSS between 2004 and 2016, arising from health conditions coded as B57, i.e. Chagas disease’ and its health consequences/impairments, defined in the Tenth Revision of the International Statistical Classification of Diseases and Related Health Problems (ICD-10) as follows: B57 (Chagas’ disease); B57.0 (acute Chagas’ disease with heart involvement); B57.1 (acute Chagas’ disease without heart involvement); B57.2 (Chronic Chagas' disease with heart involvement); B57.3 (Chronic Chagas' disease with digestive system involvement); B57.4 (Chronic Chagas' disease with nervous system involvement); B57.5 (Chronic Chagas' disease with other organ involvement); K23.1 (megaesophagus in Chagas’ disease); and K93.1 (megacolon in Chagas’ disease). 

The following variables were analyzed: sex (male; female), age at onset of Chagas’ disease (in years) and age at onset of disability (in years), time elapsed between disease and disability (in years), age group (in years: up to 29; 30-49; 50-59; 60 or over), zone of residence (urban; rural), geographic macro-region of residence (North; Northeast; Southeast, South; Midwest), type of benefit received (social assistance; social security), year the benefit was granted (2004 to 2016), and category of social security membership (self-employed; special insurance; employee; unemployed; other). The ‘sex’ variable adhered to the biological distinction as recommended by the Sex and Gender Equity in Research guidelines.^19^

Data completeness and consistency analysis was performed before the inferential statistical analysis. The data were assessed descriptively, using percentages (for categorical variables), measures of central tendency and dispersion (for numerical variables). Blank fields or fields with discrepant values were checked: those that could be corrected by analyzing other variables were changed; and the remainder were discarded from the specific analysis.

We used Epi Info 7.2.2 (CDC, Atlanta, USA) to perform statistical analyses. We calculated means and standard deviations (SD) for the continuous variables. The frequencies of social assistance, social security and total benefit grants were adjusted for each 100,000 inhabitants, for the purpose of comparing the mosaic maps of the geographic macro-regions. We used the Brazilian Institute of Geography and Statistics (Instituto Brasileiro de Geografia e Estatística - IBGE) population estimate for the year 2016, applying the following formulae: 

SAB = (Total SAB granted in the GM/EP of the GM) x 100,000 inhab.

SSB = (Total SSB granted in the GM/EP of the GM) x 100,000 inhab.

TB = (TB granted in the GM/EP of the GM) x 100,000 inhab.

where: SAB = social assistance benefits; GM = geographic macroregion; EP = estimated population; SSB = social security benefits; TB = total benefits (social assistance and social security).

With regard to data spatialization, we used the QGIS 2.18 Geographic Information System in order to prepare the mosaic maps. The analytical units were the five Brazilian macro-regions.

Association between the independent variables and the type of benefit received was analyzed by logistic regression. Sex, age group, zone of residence and geographic macro-region were used as independent variables, taking the type of benefit received as the dependent variable (social security benefit was used as the reference category). In the crude models, each independent variable was analyzed individually in relation to the dependent variable. The adjusted model included all four independent variables simultaneously. The reference categories for the analyses were those with the lowest frequency of social assistance benefits. The results of the logistic regression are presented as odds ratios (OR) and 95% confidence intervals (95%CI).

The study project was approved by the Universidade Federal de Uberlândia Human Research Ethics Committee, as per Opinion No. 1.560.139, issued on May 17, 2016.

## Results

**Figure 1:**
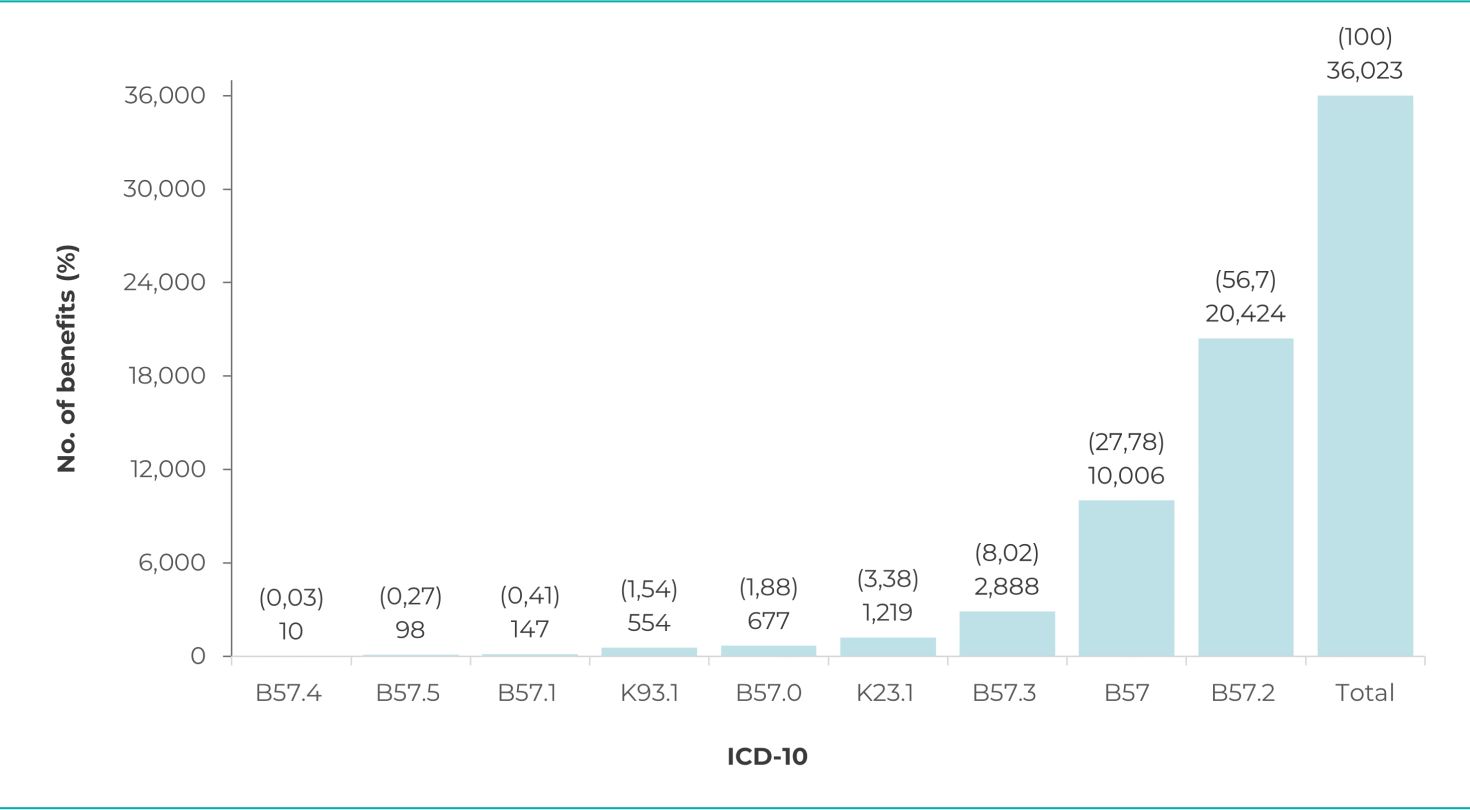
Distribution of total benefits (social security and social assistance) granted to people with Chagas’ disease, according to the Tenth Revision of the International Statistical Classification of Diseases and Related Health Problems (ICD-10), Brazil, 2004-2016

Legend: B57 = Chagas’ disease (unspecified); B57.0 = Acute Chagas’ disease with heart involvement; B57.1 = Acute Chagas’ disease without heart involvement; B57.2 = Chagas’ disease (chronic) with heart involvement; B57.3 = Chagas’ disease (chronic) with digestive system involvement; B57.4 = Chagas’ disease (chronic) with nervous system involvement; B57.5 = Chagas’ disease (chronic) with other organ involvement; K23.1 = Megaesophagus in Chagas’ disease; K93.1 = Megacolon in Chagas’ disease.

Between 2004 and 2016, 36,023 benefits were granted, mostly to males (22,503; 62.5%) and people residing in urban areas (24,155; 67.0%). The chronic form of Chagas’ disease with heart involvement predominated (20,424; 56.7%) and only 824 (2.3%) grants were due to acute forms of the disease (Figure 1). Most of the benefits were granted to adults in the 30-49 (14,794; 41.1%) and 50-59 (15,400; 42.7%) age groups.

**Figure 2:**
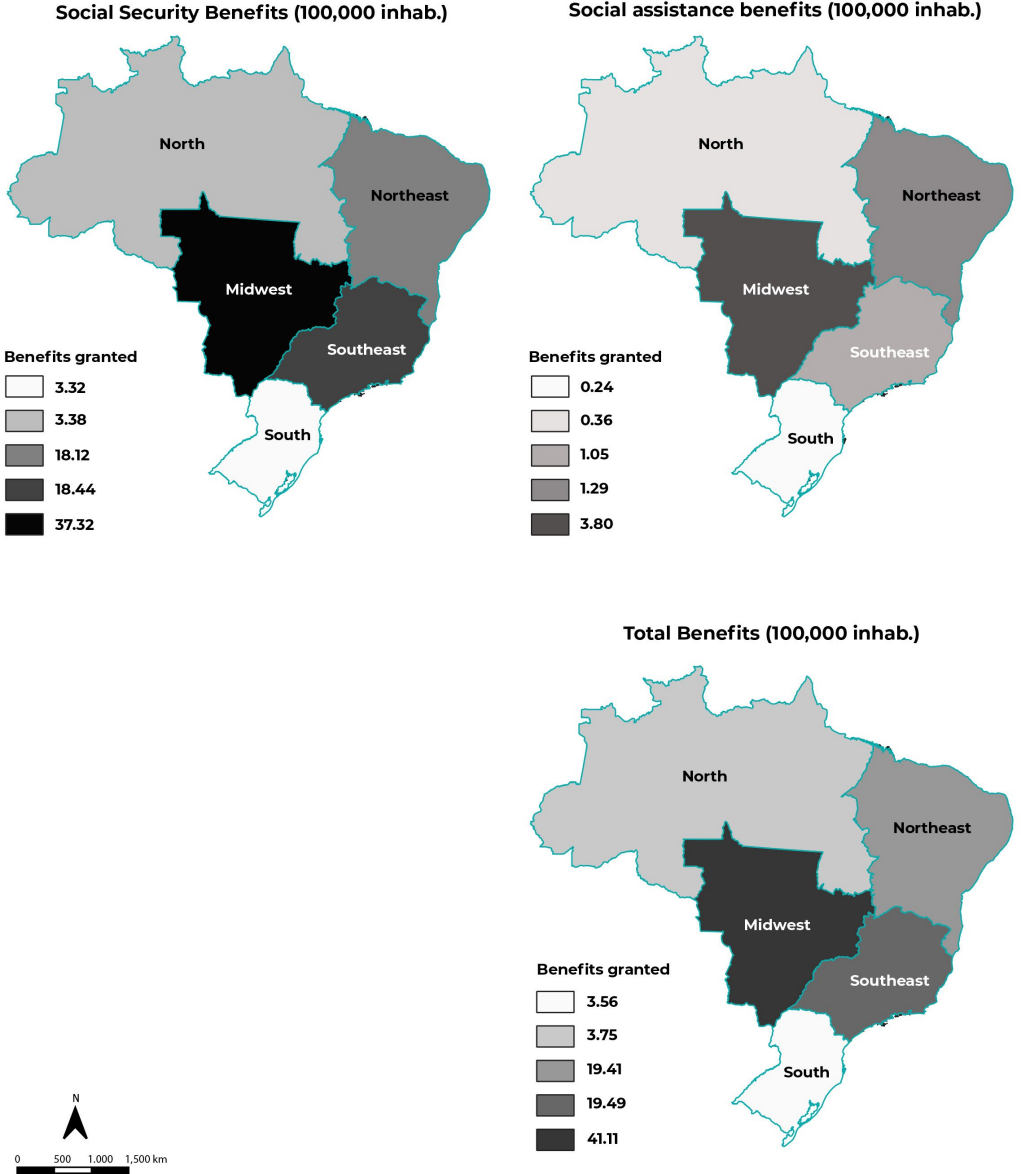
Distribution of benefits granted (per 100,000 inhabitants) to people with Chagas’ disease, by geographic macro-region and type of benefit, Brazil, 2004-2016

Total granting of benefits was predominant in the Southeast region (16,828; 46.7%). However, analysis of the granting of benefits per 100,000 inhabitants indicated that this indicator was highest in the Midwest region with regard to social security benefits (37.32/100,000 inhabitants) and social assistance benefits (3.80/100,000 inhabitants) (Figure 2).

The most granted benefit was the temporary incapacity benefit (23,417; 65.0%), followed by the permanent incapacity retirement pension (10,116; 28.1%) and the disability benefit (2,367; 6.6%). The average age of the beneficiaries of permanent incapacity retirement pensions was 52 (SD = 9.0) years. Among the forms of social security system membership, special insurance (11,597; 32.2%), employed (8,411; 23.4%), unemployed (7,495; 20.8%) and self-employed (6,147; 17.1%) were the most frequent. Special insurance was the predominant form of membership (11,597; 97.7%) among those living in rural areas.

On average, individuals had been ill for 4.7 (SD = 7.3) years prior to work incapacity. Mean age at onset of Chagas’ disease was 44 (SD = 10) years, while mean age at onset of work incapacity was 49 (SD = 9.2) years.

**Table 1:** Distribution of Brazilian social security and social assistance beneficiaries with Chagas’ disease, according to individual and demographic characteristics, Brazil, 2004-2016

Characteristics	Social security benefits	Social assistance benefits	Total	OR_b_^a^(95%CI)^b^	OR_a_^c^(95%CI)^b^
	n (%)	n (%)	n (%)		
**Sex**					
Male	21,373 (95.0)	1,130 (5.0)	22,503 (100.0)	1.00	1.00
Female	12,283 (90.8)	1,237 (9.2)	13,520 (100.0)	1.9 (1.7;2.1)	2.0 (1.8;2.1)
**Age group (in years)**					
≤29	761 (92.5)	61 (7.5)	822 (100.0)	1.5 (1.1;1.9)	1.5 (1.1;1.9)
30-49	14,032 (94.8)	762 (5.2)	14,794 (100.0)	1.00	1.00
50-59	14,367 (93.3)	1,033 (6.7)	15,400 (100.0)	1.3 (1.2;1.4)	1.2 (1.1;1.3)
≥60	4,496 (89.3)	511 (10.7)	5,007 (100.0)	2.1 (1.9;2.3)	1.6 (1.3;1.7)
**Zone of residence**					
Rural	11,855 (99.9)	13 (0.1)	11,868 (100.0)	1.00	1.00
Urban	21,801 (90.2)	2,354 (9.8)	24,155 (100.0)	98.5 (57.1;169.9)	134.9 (78.0;233.2)
**Geographic macro-region**					
Southeast	15,924 (94.6)	904 (5.4)	16,828 (100.0)	1.00	1.00
North	599 (90.3)	64 (9.7)	663 (100.0)	1.9 (1.4;2.5)	2.8 (2.1;3.7)
Northeast	10,313 (93.4)	732 (6.6)	11,045 (100.0)	1.3 (1.1;1.4)	2.9 (2.5;3.1)
South	976 (93.1)	72 (6.9)	1,048 (100.0)	1.3 (1.0;1.7)	1.3 (1.0;1.7)
Midwest	5,844 (90.7)	595 (9.3)	6,439 (100.0)	1.8 (1.6;2.0)	1.6 (1.5;1.8)

a) OR_b_: Crude *odds ratio*; b) 95%CI: 95% confidence interval%; c) OR_a_: *Odds ratio* adjusted for sex, age group, zone of residence and geographic region.

Females were twice as likely to receive social assistance benefit (OR = 2.0; 95%CI 1.8;2.1) (Table 1). Granting of social assistance benefits was also positively associated with living in urban areas (OR = 134.9; 95%CI 78.0;233.2) compared to rural areas. The odds of social assistance benefits being granted were highest in the ‘up to 29 years’ and the ‘60 years and over’ age groups (OR = 1.5; 95%CI 1.1;1.9 and OR = 1.6; 95%CI 1.3;1.7, respectively), when compared to the 30-39 age group (Table 1). The national macro-regions with the highest odds of granting social assistance benefits were the Northeast (OR = 2.9; 95%CI 2.5;3.1) and the North (OR = 2.8; 95%CI 2.1;3.7) (Table 1).

**Figure 3:**
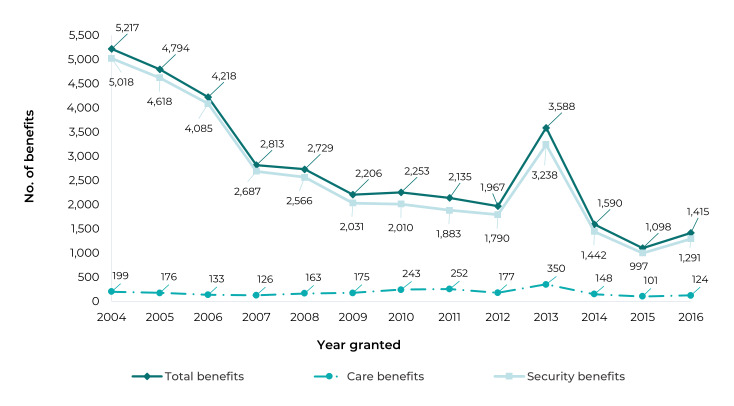
Benefits granted to people with Chagas’ disease, by year granted, Brazil, 2004-2016

The greatest number of total benefits was granted in 2004 (5,217; 14.5%), after which it decreased gradually until 2016 (1,415; 3.9%), with the exception of 2013 (Figure 3). 

## Discussion

The analysis showed that in the period between 2004 and 2016, Brazilian social welfare beneficiaries with Chagas’ disease were mainly male, lived in urban areas, lived in the Southeast region, had the chronic heart involvement form of the disease, and were between 50 and 59 years old. The granting of social assistance benefits was associated with living in urban areas, living in the Northeast macro-region, being female and being 60 years old or over.

Chagas’ disease is widely distributed over the American continent, where the *T. cruzi* parasite is considered to be endemic.^3^ In Brazil, 3,222 cases of Chagas’ disease were confirmed between 2004 and 2016,^20^ and during that period, a total of 36,023 benefits related to the disease were granted by the social welfare service. This discrepancy between the number of cases notified and the number of benefits granted can be attributed to two factors. One is that compulsory notification of chronic cases of Chagas’ disease throughout all of Brazil only became mandatory in 2020.^18^ As such, cases of the disease reported on the Notifiable Health Conditions Information System (Sistema de Informação de Agravos de Notificação - SINAN) during the study period only accounted for acute Chagas’s disease cases. Moreover, given the slow and progressive evolution of the disease over the years, it is likely that most benefits were granted to individuals who had been infected in the past.^21^ The low percentage of acute cases corroborated in this study this statement.

Chagas’ disease is mostly diagnosed in its chronic stage,^6^ when the severity of its manifestations has a strong impact on the health of the workers, leading to work incapacity and, consequently, to a greater number social security and social assistance benefits being granted. Chronic Chagas’ cardiopathy is of particular relevance because it is the most frequent clinical form of Chagas’ disease and has high potential for causing incapacity.^22^

Mean age of Chagas’ disease onset and onset of work incapacity among affected individuals was below 50 years. In a study that used data from the Global Burden of Disease Study 2016 for the period 1990 to 2016, 141,640 DALYs were estimated to have been lost due to Chagas’ disease in 2016, which corresponds to a reduction of 36.7% in DALYs in comparison with the estimates of this indicator for the year 1990.^23^ One DALY means one year of healthy life lost, taking into account premature deaths and years lived with incapacity.^2^ These data prove that, despite the reduction of cases experienced in recent decades, Chagas’ disease remains an important cause of morbidity, mortality and disability in Brazil.

A higher frequency of benefits (social security and social assistance) was found for males. However, the odds of receiving social assistance benefits were highest among females. Social security coverage for women under the General Brazilian Social Security System, which is the system analyzed in this study, has always been lower than social security coverage for men, even after the changes that took place in the 1990s, when women’s share in the labor market increased significantly, and continued to grow in the 2000s.^24^ Women are the majority among workers without a labor card, unpaid workers, and workers who produce items for their own consumption, these being facts that are reflected in the differences found in this study between the sexes in percentages of social security benefits granted.^24^ However, women are more representative than men among the contributors to Statutory Social Security Systems, since they are the majority among Health and Education employees.^25^

In the first half of the 20^th^ century, in Brazil, most cases of Chagas’ disease were concentrated in rural areas, the natural *habitat* of the disease vectors. The industrialization of the country, the growth of cities, and the rural exodus caused individuals infected in the past, in rural areas, to migrate to urban areas, leading to a new epidemiological context.^6^ The greater frequency of social welfare benefits for people with chronic Chagas’ disease living in urban areas reflects this change in the profile of the disease. Furthermore, rural dwellers have less access to social and health services and therefore the reduced number of claims for benefits granted to residents in these areas may be associated with lack of knowledge about their social rights.^6^

The rural population still presents characteristics that differ greatly from the urban population, and the percentage of individuals in the “special insurance” category of the social security system is an example of this. This category accounts for smallholders and artisanal fishermen, who perform their work individually or as family workers, without employees.^25^ The family economy system is understood to be activities in which the work of the family members is indispensable for their own subsistence and for the socioeconomic development of the family nucleus.^25^ In this way, social vulnerability (individual and family) is mitigated, which also results in fewer benefits being granted, especially social assistance benefits.

With the decrease in the incidence of Chagas’ disease in recent decades and the aging of the population that has been infected in the past (therefore, with greater chances of developing clinical manifestations), greater occurrence of the disease is expected among adults and the elderly.^6^ Indeed, most of the beneficiaries were adults aged 30 to 59 years; the elderly accounted for less than 15%. This low frequency among the elderly may be associated with the high mortality of the disease in older age groups. A study conducted in Brazil, with data from 2000 to 2019, showed a direct relationship of the Chagas’ disease mortality rate with increasing age groups, reaching the maximum value among individuals aged 80 years or older (42.3/100,000 inhab.).^26^

In the present study, the approach to the granting of social assistance benefits was restricted to social assistance for people with disabilities related to Chagas’ disease, which are more associated with the younger and more elderly age groups. Considering the slow and chronic progression of the disease, incapacity to work among individuals up to 29 years of age draws attention. Those who are eligible for social assistance benefits demonstrate a situation of significant vulnerability, characterized by the inability to have the means to provide for their own maintenance, work incapacity caused by the disease (with greater social impact in the younger age group) and precarious family conditions (income per person in the family group less than 1/4 of the minimum wage). In the older age groups, the chronicity of Chagas’ disease, associated with comorbidities found in the aging process, increases the risks for individuals and the demands on the health system, representing a great challenge for the Brazilian National Health System.^6^

The Southeast region had higher frequencies of benefits granted, both with regard to social security and social assistance benefits. Besides being Brazil’s most populous and urbanized macro-region, it has higher human development indexes, so that its resident population is more able to contribute to social security; and is better informed as to existing and relevant welfare rights.^27^ However, when the benefits were analyzed according to population, the Midwest region had the highest number of benefits granted, both with regard to social assistance and social security. This geographical distribution coincides with an area considered to have high endemicity and intense vector transmission in past decades, an example being the state of Goiás, where there has been intense migration of people from endemic rural areas to urban centers, such as the Federal District.^6^ ^,^ ^26^ ^,^ ^28^ Greater odds of social assistance benefits being granted were associated with the North and Northeast macro-regions, suggesting greater social vulnerability of their populations.^27^

Temporary incapacity benefit was the type most frequently granted, followed by permanent incapacity retirement pension. This shows that the majority of beneficiaries with chronic Chagas’ disease contributed to social security and collaborated with country’s labor force until their temporary or permanent work incapacity was recognized. Similar research carried out in Brazil on social welfare and AIDS, for the same period as this study, found that of 99,369 beneficiaries, 26.5% received welfare benefits and 51% were unemployed.^11^ ^,^ ^12^

There was a progressive reduction in the number of benefits granted to sufferers of chronic Chagas’ disease in Brazil in the period analyzed. This number followed the epidemiological profile of the disease in the national territory. With the control and reduction of intra-household transmission by vectors and the control of transmission by blood transfusion, the incidence of the disease has reduced drastically in recent decades in Brazil.^6^ The consequences of these actions are evident in the low percentages of benefits granted to younger age groups which, together with Chagas’ disease mortality among individuals aged 50 to 59 years and older, has contributed to the reduction of its impact on the social security and social assistance system.^26^

The peak in the number of benefits granted in 2013 is also noteworthy. Outbreaks of acute Chagas disease cannot justify this increase, since in general they do not affect more than a few dozen people, and not all of them are beneficiaries of social welfare or become unable to work.^6^ Furthermore, most of these outbreaks have developed in the state of Pará, which, in this study, had one of the lowest percentages of benefits granted.^6^ Peaks in the number of benefits granted can also occur as a result of beneficiaries themselves telling other people about their social security and social assistance claims that have been met, or by the work of lawyers who specialize in certain types of social security cases and request them, successively, for a group of people.

The social security reform in 2019 resulted in an important reduction in the amounts to be received for permanent disability retirement benefits and temporary disability aid.^29^ Considering that a large number of people with chronic Chagas’ disease are eligible for these benefits, an increase in the social vulnerability of this hard-hit group is expected. Moreover, with the advent of the COVID-19 pandemic, access to social welfare benefits has been hampered by the lack of face-to-face administrative assistance and by the need to use digital technologies to solve pending issues related to applying for benefits, since part of the population does not have internet access and/or mastery of these tools.^30^ This reality constitutes an important element of the reduction found in the granting of benefits, and needs to be considered with regard to future cases.

Uncertainty about data reliability - inherent to any study using secondary data - is a limitation to be considered in this study. The lack of previous research on the subject made comparisons impossible and can also be pointed out as a limitation. In addition, our analysis did not include people with Chagas’ disease who were not registered with the social security system and/or those without the basic requirements to be entitled to social assistance benefits. When they work informally, they remain outside the social welfare system, in relation to social security and social assistance, and therefore, this condition can lead to underestimation of the number of workers affected by the disease, constituting another important limitation of our study.

The analysis of the data we collected fills a gap in knowledge about social welfare and Chagas’ disease in the Brazilian context. Despite the reduction in the incidence of the disease in the country, cases infected in the past continue to significantly impact the Brazilian health, social security and social assistance systems, especially among the adult and elderly populations. Public health policies aimed at the chronically ill should be implemented, in order to provide a better quality of life for those who have the disease and contribute to the stabilization, delay or even clinical improvement of cases, and consequently, to reduce the labor impact of Chagas’ disease in Brazil.
